# Understanding Adolescents’ Experiences of Cyberbullying in Jordan: Evidence From a Narrative Perspective

**DOI:** 10.2196/92120

**Published:** 2026-06-25

**Authors:** Anwar M Eyadat, Maha M Albdour, Hanan A Abusbaitan, Vipavee Thongpriwan, Young Ik Cho, Amani Mobarki, Jovan Gwon

**Affiliations:** 1 Department of Community and Mental Health Faculty of Nursing The Hashemite University Zarqa Jordan; 2 College of Nursing Wayne State University Detroit, Michigan, MI United States; 3 School of Nursing Oakland University Rochester, MI United States; 4 School of Nursing University of Wisconsin–Milwaukee Milwaukee, WI United States; 5 Joseph J Zilber College of Public Health University of Wisconsin–Milwaukee Milwaukee, WI United States; 6 College of Nursing and Health Sciences Jazan University Jazan Saudi Arabia; 7 School of Nursing University of Minnesota Minneapolis, MN United States; 8 Masonic Cancer Center University of Minnesota Minneapolis, MN, MN United States

**Keywords:** adolescents, consequences, cyberbullying, online harassment, victimization

## Abstract

**Background:**

Based on its nature, cyberbullying is expected to be frequent and prevalent because of the continuity of technological advancement over time. This change results in more victims and is challenging to detect, negatively affecting their health. Given that adolescents in Jordan face a high rate of cyberbullying, it is essential to understand how this experience affects them.

**Objective:**

This study aims to explore individual experiences and perspectives on cyberbullying victimization among adolescents to intervene and reduce future incidences of cyberbullying.

**Methods:**

We based our analysis on a cross-sectional study investigating cyberbullying and its mental health consequences among 400 students aged 14-17 years from public schools in central and northern Jordan. These respondents were asked to answer 3 open-ended questions describing their experiences with cyberbullying if they experienced cybervictimization as either victims or bully-victims, resulting in 240 responses. Thematic analysis was then used to interpret patterns of shared meaning across participants’ narratives related to cyberbullying experiences in cyberspace.

**Results:**

A total of 3 key themes and several subthemes emerged from this study: (1) effects of cyberbullying, (2) challenges in overcoming its consequences, and (3) elements influencing the severity of cyberbullying experiences.

**Conclusions:**

The findings offer valuable insights for creating safer online environments and reducing cyberbullying’s psychological and social harms through appropriate interventions.

## Introduction

Cyberbullying is defined as the intentional and repeated use of information and communication technologies to harm, harass, or humiliate others [1]. While the concept of bullying itself has long been studied, the rise of information and communication technologies has shifted many aggressive behaviors into digital spaces. The foundational work of Olweus [2] on traditional bullying remains relevant; however, cyberbullying differs significantly because of its anonymity, potential for wide audience reach, and persistence [3,4].

Adolescents are particularly vulnerable to cyberbullying due to their developmental stage, marked by rapid emotional, social, and cognitive changes [5,6]. According to Bronfenbrenner’s Ecological Systems Theory, adolescents interact with multiple individual, family, peer group, and environmental factors that can all influence their experiences with cyberbullying [7]. As they form their identities and social competencies, they become more susceptible to peer pressure, social comparison, and emotional distress, increasing the risk of being both victims and perpetrators of cyber aggression [8,9]. Within this context, other findings suggest that extended online engagement, particularly during nonschool hours, combined with the use of anonymous or fake profiles, significantly increases the risk of cyberbullying [10]. The anonymity afforded by fake identities appears to lower social accountability and facilitate harmful behaviors, positioning such profiles as key risk factors in the digital ecology of adolescent cyberbullying [10].

Globally, cyberbullying prevalence varies depending on age group, data collection methods, and cultural context. In the United States, studies report adolescent victimization rates ranging from 3% to 72% and perpetration rates ranging from 1% to 41% [11]. Another national American sample of 1034 preadolescents aged 9-12 years completed an online survey to report their experience with cyberbullying. Of them, 21% have been exposed to cyberbullying as a target, aggressor, or witness to cyberbullying [12]. In Malaysia, 31.6% of adolescents reported recent victimization, and 20.9% reported perpetration [13]. In Indonesia, approximately 80% of seventh-grade students reported experiencing occasional cyber victimization [14]. In Saudi Arabia, the rate was 42.8%, with boys more affected than girls [15]. In Jordan, few studies exist, but moderate to high prevalence has been reported, often linked to educational level and academic performance [16-18]. These findings suggest that cyberbullying is a widespread issue, but one that requires culturally contextualized understanding.

Cyberbullying has serious psychological consequences, particularly for adolescents. Victims often experience anxiety, depression, low self-esteem, and social withdrawal [19,20]. Cyberbullying differs from traditional bullying in its ability to invade the victim’s private space continuously, and the anonymity of aggressors intensifies its effects [4]. The diathesis-stress model of psychopathology posits that psychological problems arise from a combination of an individual’s experience of stressful events and their inherent susceptibility to developing the disorder [21]. This model helps explain how individual vulnerabilities (eg, impulsivity and low self-regulatory efficacy) interact with environmental stressors such as cyberbullying to produce mental health symptoms [22]. Limited coping skills, a lack of social support, and the ubiquity of digital access can exacerbate the impact [3].

Despite growing research on cyberbullying, many studies focus on prevalence and institutional responses, often overlooking the subjective experiences of adolescent victims. A deeper comprehension of the context of bullying and its effects on young people is required [23]. There remains a critical gap in understanding how victims personally experience and cope with cyberbullying, as well as the long-term psychological, emotional, and social repercussions of this type of behavior. This gap is particularly critical in regions such as the Middle East, where digital behaviors intersect with unique social and cultural norms [24]. In Jordan, youth face distinct challenges related to family expectations, gender norms, and limited digital literacy resources [25,26]. These factors may shape how cyberbullying is perceived, reported, and addressed. Understanding adolescents’ lived experiences in this context is essential for developing effective, culturally grounded interventions. Since cyberbullying can be prevented through several interventions and programs [27], current interventions might not be deep enough to address victims’ particular struggles if victims’ voices are not included, which could result in an insufficient support network.

Therefore, this study aims to explore the lived experiences of Jordanian adolescents who have been victims of cyberbullying, focusing on their emotional reactions, coping mechanisms, and self-perceptions. This victim-centered approach is grounded in the principles of trauma-informed care, which emphasizes safety, empowerment, and recognition of the long-term psychological impact of trauma, especially in adolescent populations [28,29]. By centering adolescents’ narratives, the study aligns with narrative psychology, which recognizes the role of personal storytelling in shaping identity, meaning-making [30], psychological growth, and event distress [31]. Furthermore, this perspective reflects the principles of participatory and context-sensitive research, which advocate for involving participants as active contributors to knowledge production and ensuring interventions are culturally and socially relevant [32]. In doing so, the study addresses a critical gap in the literature and contributes valuable insights for the design of culturally grounded, psychologically responsive cyberbullying interventions in the Jordanian context.

Since this study is likely grounded in exploratory and descriptive goals rather than hypothesis testing, several exploratory questions were provided to align with the narrative and qualitative nature of the study, as follows:

- How do adolescents who have experienced cyberbullying describe its effects?
- What factors do adolescents who have experienced cyberbullying perceive as worsening the impact of cyberbullying on their well-being?
- What ongoing challenges to recovery from cyberbullying do adolescents who have experienced cyberbullying report?

## Methods

### Ethical Considerations

Institutional Review Board approval was obtained first from the University of Wisconsin-Milwaukee (#24.112), followed by approval from the Jordan Ministry of Education (#4218) and school principals. Participants were informed of their right to withdraw at any time without consequences and of the confidentiality of their data. Parental consent and student assent were secured before data collection. The procedures used in this study adhere to the tenets of the Declaration of Helsinki.

### Design and Setting

This is a qualitative descriptive study embedded within a cross-sectional survey aimed at examining the prevalence of cyberbullying and investigating its mental health impacts among adolescents and whether gender may moderate the correlation between cyberbullying and mental health. The current qualitative analysis was not explicitly designed to investigate gender differences in narrative content. The main objective was to investigate common experiences and significant themes concerning the effects of cyberbullying on adolescents instead of performing a comparison among demographic subgroups. Using a qualitative descriptive approach provides a practical method for revealing the facts of a phenomenon without requiring an in-depth focus on meaning, as in other qualitative methods such as phenomenology, especially among adolescents [33]. A total of 8 governmental schools in central and northern Jordan were selected for their accessibility and the availability of teachers to facilitate data collection. These schools accommodate a large student population due to mandatory primary education laws [34], with Amman and Irbid, the most populous cities, facilitating recruitment.

### Participants, Sample, and Instrument

Participants were male and female adolescents aged 14-17 years in grades 9-11 from public schools in central and northern Jordan. A multistage stratified cluster sampling method was used to select geographical areas, education directorates, schools, and classes. Students diagnosed with uncontrolled mental illness, intellectual disabilities, or narcotic drug use were excluded because of potential impacts on mental health and cyberbullying involvement. School representatives (eg, school principals or teachers) were asked whether students in the selected classes had any detected exclusion conditions that needed to be addressed to ensure the exclusion criteria were met. Also, the exclusionary conditions (chronic illness and untreated psychological disorder) were explained in the consent form so that participants or guardians could decide. Accordingly, no one was excluded.

A total of 400 participants completed a survey consisting of several sections. Participants’ involvement in cyberbullying was assessed using the Revised Cyber Bullying Inventory–II (RCBI-II) Questionnaire [35], which includes 10 statements presented in 2 parallel columns, and participants must respond from the viewpoints of both the bully and the victim in the last 6 months. Each item is rated on a 4-point Likert scale reflecting the frequency of behaviors. Based on participants’ scores, individuals were categorized into involvement groups. A “bully-victim” was operationally defined as a participant who scored >10 points in both the cyberbullying and cybervictimization sections. In contrast, “pure victims” were those who scored >10 points on cybervictimization only without endorsing any perpetration behaviors, whereas “pure perpetrators” reported perpetration only (scored >10 points on the cyberbullying section). This classification approach is consistent with prior studies using the RCBI-II, allowing differentiation among distinct roles in cyberbullying involvement.

The last section of the survey asked participants to complete it only if they had experienced victimization. As a result, 240 participants (identified as victims or bully-victims based on the RCBI-II) answered 3 additional questions narratively about their online victimization to understand the impact of cyberbullying on them. Those questions were as follows: “What was the effect of the cyberbullying involvement you have had?” “What do you think makes the effect of experiencing cyberbullying worse on you?” and “What are the factors that determine the severity of cyberbullying impacts?” Each of these questions was intended to answer the previously provided research questions. Open-ended survey responses were selected for their ability to enable participants to convey personal experiences in a flexible, anonymous, comfortable, and less intrusive way compared with semistructured interviews or focus groups, which was especially fitting given the sensitive nature of the subject and the adolescent study group. This approach also allowed the inclusion of diverse, large samples within the limits of time, resources, and accessibility, which might not have been practical with interviews or focus groups.

### Procedure

Data were collected between January and February 2024. Students were briefed on the study’s purpose, procedures, voluntary participation, confidentiality, and withdrawal rights during the first interview. Parental consent and student assent were secured before data collection. Consent forms were distributed during parent-teacher conferences and sent home for signature. School representatives (eg, school principals or teachers) were asked to send the recruitment flyer to parents who did not attend the conferences, reach out to them, explain the study’s purpose and procedures, and send the consent forms. Data were collected individually using paper-and-pencil questionnaires in private rooms by researchers and assistants administering the questionnaires, taking 30-40 minutes per student. Personal incentives were provided upon completion, but they were simple, such as pens and notebooks, to avoid any bias in participation or responses. Consent forms and questionnaires were entered into Qualtrics via University of Wisconsin-Milwaukee access. At the same time, hard copies were kept anonymous and securely stored in a locked file cabinet in the researcher’s office in Jordan.

When using mental health, behavioral, and quality-of-life assessments, the Committee on the Use of Humans as Experimental Subjects evaluates the merits and drawbacks of addressing these topics with study participants [36]. It assists researchers in determining whether to include mental health-related inquiries and devising risk mitigation plans. Unintentional identification may occur when assessing quality-of-life issues like sadness, anxiety, or stress, prompting the Committee on the Use of Humans as Experimental Subjects to recommend a “safety plan” for populations at elevated risk [36]. This plan involved assessing the immediacy of risk by qualified researchers on “Social and Behavioral Research Best Practices for Clinical Research,” providing staff training on intervention procedures, and offering participants a resource referral flyer because the data would be anonymous; the flyer included mental health resources, crisis intervention services, or hotline information, depending on the type of risk. After completing data collection, none of the participants required intervention under this protocol.

### Data Analysis

#### Overview of the Analytic Process

Descriptive statistics, including means, SDs, percentages, and frequencies, were used to describe participants’ demographic characteristics. Then, a thematic analysis strategy was used as a structured and flexible framework to detect, analyze, and interpret patterns of shared meaning across participants’ narratives of cyberbullying experiences in cyberspace [37]. Braun and Clarke’s [38] 6-phase framework for thematic analysis was specifically used to analyze the data and is one of the most clearly defined approaches to performing reflexive thematic analysis to describe and summarize data and to offer a range of higher-level inductive or deductive analyses of small or large datasets [39]. The phases are familiarization, coding, initial theme generation, theme development and review, theme definition and naming, and writing up [38]. This framework was suited to this study’s aim of exploring the lived experiences of Jordanian adolescents who have been victims of cyberbullying, concentrating on their emotional reactions, coping mechanisms, and self-perceptions. The application of each phase is described in the following subsections.

#### Phase 1: Familiarization With the Data

This stage involves examining and reexamining data to gain a deeper understanding of the specifics and the broader context of the data, typically beginning with an emphasis on the more evident semantic interpretations before progressing to an analytical engagement with the data [38]. Applying this, participants’ responses were translated from Arabic to English using a forward-backward strategy by 2 bilingual translators proficient in both English and Arabic, with knowledge of health care and qualitative research terminology. The first translator translated the Arabic responses into English. The translator and research team discussed and compared the translated versions to create a unified, agreed-upon English version.

Then, backward translation into Arabic was conducted by a second translator who was blinded to the original Arabic version to ensure accuracy and evaluate language clarity. The back-translated versions were compared with the initial responses to detect inconsistencies, ambiguities, or loss of significance. Careful emphasis was placed on maintaining the semantic, cultural, and contextual significance of terms associated with cyberbullying experiences in the Jordanian sociocultural framework. The first author read and reread the transcripts to gain an in-depth understanding of the participants’ accounts while noting preliminary impressions and recurrent expressions related to emotional reactions, coping strategies, and perceptions of online interactions.

#### Phase 2: Coding

This phase starts by organizing data systematically and meaningfully, reducing large amounts into smaller, more meaningful segments. Coding methods can vary, but one approach is to address the research question, which yields theoretical rather than inductive analysis. Thereby, following open coding, which means that we do not have preset codes but rather a coding process [40], we assigned codes to every segment of data that pertained to or highlighted something significant regarding our research question. To enhance engagement with participants’ responses, the original coding was carried out manually by the first author, who identified meaningful text units, essential elements, related phrases, and patterns that reflected the research aim, condensed them, arranged them according to similarities and differences, and lastly assigned codes that possess characteristics such as “anxiety and fear,” “stress and pressure,” and “social agreement.”

A selection of the transcripts was independently reviewed by the senior author, who also provided interpretive comments on the coding scheme and thematic organization to ensure reliability. Through cooperative interpretation and consensus building, investigator triangulation was used to reinforce trustworthiness, even though formal intercoder reliability metrics were not computed. Two authors reviewed transcripts and coded data independently to minimize individual interpretive bias. Inconsistencies in coding and theme understanding were addressed through ongoing discussions and agreement. To enhance analytical rigor and minimize possible coder bias, reflexive memoing and peer debriefing were also used to record researchers’ beliefs, analytical choices, and thoughts regarding possible impacts on data interpretation. The researchers were mindful of how their expertise and prior knowledge of cyberbullying research might influence their interpretations; therefore, they engaged in ongoing critical reflection throughout coding and theme development. Emphasis was placed on reducing power dynamics by highlighting voluntary participation, ensuring confidentiality, and allowing participants to skip questions or withdraw at any time.

#### Phase 3: Search for Themes

A theme is a manner that reveals something meaningful or intriguing concerning the data and/or research inquiry [40]. To align with that, the codes were checked for conceptual uniformity and grouped into candidate themes developed by observing recurring patterns in the data. For instance, following connected codes “anxiety and fear,” “stress and pressure,” and “emotional distress” were assorted into the psychological effects of cyberbullying theme. While codes reflecting “facing the uncomfortable,” “disconnection,” and “invisible perpetrators” formed a theme called challenges in overcoming cyberbullying consequences. Thematic analysis was conducted iteratively, and codes were contrasted across responses until no new themes emerged, showing a degree of thematic adequacy suitable for this research design. Despite the responses being relatively brief, the substantial sample size (n=240) provided a wide range of viewpoints, enabling the identification of consistent trends and prevalent themes.

#### Phase 4: Reviewing Themes

The identified themes were then reviewed to enhance clarity and coherence. Some overlapping codes were merged, and all themes were checked against the data to verify their reflection of a deeper understanding of participants’ viewpoints and experiences with cyberbullying. When suitable, subthemes were created to encapsulate detailed yet consistent patterns of significance within overarching themes. Subthemes were used purposefully to deepen analysis and were not regarded as hierarchical classifications or subcategories. This iterative process enhanced the validity and clarity of the thematic structure.

#### Phase 5: Defining and Naming Themes

The final thematic structure included 3 major themes: effects of experiencing and being involved in cyberbullying (psychological and social effects), challenges in overcoming consequences of cyberbullying experiences (anonymity of perpetrators, threat and exposure context, and public exposure of incidents), and elements influencing the severity of cyberbullying experiences (individual vulnerability, social response, and digital environment). All themes and subthemes were explicitly outlined to highlight a unique facet of adolescents’ experiences of cyberbullying in digital environments, focusing on the interaction between emotional distress, social relationships, and situational influences. During this process, analytic choices were influenced by a focus on meaning-based interpretation rather than frequency or classification.

#### Phase 6: Producing the Report

In the final phase, the results were presented using themes and subthemes, which were synthesized into a coherent narrative supported by illustrative verbatim quotations that reflected the variation and depth of experiences, in parallel with Braun and Clarke’s [38] reflexive thematic analysis. This framework provides a rich understanding of the psychological, social, and technological aspects of cyberbullying as experienced by adolescents. The findings were interpreted within the framework of adolescent developmental psychology and the sociocultural context of Jordan.

## Results

### Descriptive Characteristics of the Participants

A total of 240 participants admitted to engaging in cyberbullying as a victim or bully-victim; of these, 47% (113/240) were females aged 14-17 years, with a mean age of 15.2 (SD 1.01) years. Approximately half were located in Amman. The distribution of grades was approximately 34.2% (82/240) in 9th grade, 32.8% (79/240) in 10th grade, and 33% (79/240) in 11th grade. Approximately 34.8% (83/240) of participants were unaware of their monthly earnings; 30% (72/240) indicated earning between JD 500-1000 (Jordanian dinars; exchange rate: JD 1=US $0.70 as of February 4, 2024), 29% (70/240) earned less than JD 500, and 6.3% (15/240) earned more than JD 1000. Concerning academic achievement, 27% (65/240) indicated an average above 90 in the previous semester, 25.8% (62/240) averaged between 80 and 89, 23% (55/240) between 70 and 79, 13.7% (33/240) between 60 and 69, and 10.5% (25/240) below 60.

All participants had internet access, and 81.5% (196/240) reported having 1-5 social media accounts, with about 64.7% (155/240) spending 1-7 hours online daily. About 51.4% (123/240) of the people who engaged in cyberbullying were victims, and 48.6% (117/240) were bully-victims. Answers to the questions assessing the bully’s identity indicated that 47.1% (113/240) of the 240 people who reported being a victim, a bully, or a bully-victim had met a bully in real life, and 44.2% (106/240) said the bully was the same gender as them. Friending the bully was the most common relationship (99/240, 41%), followed by someone in the school (46/240, 19.2%), strangers (42/240, 17.5%), former friends 29/240, 12.1%), and chat room users (24/240, 10%), respectively. Three key themes and 8 subthemes emerged from this study: (1) effects of experiencing and being involved in cyberbullying, (2) challenges in overcoming consequences of cyberbullying experiences, and (3) elements influencing the severity of cyberbullying experiences (Table 1).

**Table 1:** Themes and subthemes related to cyberbullying experiences.

Theme	Subthemes
Effects of experiencing and being involved in cyberbullying	Psychological effects (eg, anxiety, emotional distress, and sleep disturbances) Social effects (eg, isolation, withdrawal, and relationship difficulties)
Challenges in overcoming consequences of cyberbullying experiences	Anonymity and sense of powerlessness (difficulty identifying perpetrators and lack of control) Threatening and exploitative nature (fear, coercion, and misuse of personal content) Public exposure and wider harm (rapid dissemination and long-term reputational impact)
Elements influencing the severity of cyberbullying experiences	Individual vulnerability and coping capacity (age, resilience, prior experiences, and self-confidence) Role of others in shaping the experience (peer, family, and institutional responses) Digital environment as an enabler (cyberbullying tactics, the purpose of cyberbullying, the number of bullies, the lack of regulations and penalties, and platform features that facilitate the spread of harmful content)

### Themes

#### Effects of Experiencing and Being Involved in Cyberbullying

##### Psychological Effects

Participants stated profound emotional consequences of being involved in cyberbullying as victims or bully-victims. Feelings of badness, leading to crying because they did not know how to deal with such an experience, were common. Further, feelings of emotional distress, anxiety, fear, stress, and pressure, and cognitive and behavioral issues were recurrently expressed. The following quotes illustrate those feelings. For anxiety and fear, a participant said, “I felt embarrassment, inferiority, ugliness, dissatisfaction with one’s body, and a feeling of not being included with other people in society.” While this quote, “I got angry and sensitive regarding any word,” is an example of stress and pressure. Finally, the following quote reflects cognitive and behavioral issues:

I felt unfortunate; I felt that I do not deserve friends, I do not trust anyone, even my friends, I have suicidal ideation because the people around me damage my heart.

Several participants reported intrusive thoughts, sleep disturbances, and a sense of helplessness. One participant noted:

I felt afraid to open my phone because I did not know what I would find next... I was thinking about that the whole night, so I was not able to sleep.

Some mentioned self-accusation and internalized shame, thinking they had somehow triggered or warranted the bullying. Such responses frequently resulted in diminished self-esteem and a negative self-image, as one participant explained: “I started to think, is there something wrong with me?!” Another participant stated, “I had bad feelings because I did not know how to deal with that person and get revenge on him... I felt like a useless and bad person in this life.”

##### Social Effects

Participants reported experiencing equally significant social effects. Many participants reported altered trust in others, becoming more cautious, withdrawing from social circles, and experiencing isolation and loneliness, along with changes in social situations, as a result of their experiences.

I remember when I posted strictly in the snap in a group, then a girl bullied me and said I am a Lier, black, and ugly; I left the group and broke the phone.

Another participant stated:

It is a bad experience to the degree that you do not want to confront others and felt embarrassment, making you isolated and do not want to see others.

Participants described being isolated from others because of feeling forsaken and depressed, which subsequently affected their academic performance, social interaction, and caused social anxiety and loneliness, especially if the perpetrator is a close person. Others described avoiding social interactions in schools or online platforms to avoid victimization. A participant described:

The experience impacted my social relationships directly... it affects social situation by being isolated from others and affects academic performance.

Another one said, “I stopped talking to my peers because I thought they were all laughing at me.” These experiences often damaged friendships and family relationships, intensifying feelings of loneliness and social alienation.

#### Challenges in Overcoming Consequences of Cyberbullying Experiences

##### Overview

This theme highlights several traits that make the cyberbullying experience terrible and hinder participants’ ability to recover from cyberbullying victimization, including knowing the identity of the perpetrator, the exploitative nature of cyberbullying, and public exposure.

##### The Anonymity and Sense of Powerlessness

The participants addressed this subtheme by mentioning 3 features that intensify the difficulty of coping with harm caused by a hidden perpetrator: a disconnected, invisible perpetrator, and facing the unconfrontable. The anonymity of the perpetrator is the most reported trait because the inability to see the perpetrator is a provocative thing, not to know who is behind the screen, so they say whatever they want without fear, freely, and make it difficult to confront them, subsequently, making it difficult to limit or control cyberbullying, especially through the distancing communication, which amplified feelings of powerlessness and fear. As one participant expressed, “not knowing who was behind the screen made me feel fear and unsafe everywhere.”

The following quote refers to facing the unconfrontable feature:

The anonymous feature of the perpetrator is terrible, so you cannot reach the perpetrator to confront and defend yourself and talk about your feelings.

The bullies cannot see the victim, then will say whatever they want without fear, freely, which represents the invisible perpetrator feature. The following quote is an example of the disconnected feature: “Lack of face-to-face contact, and the distance interaction makes one feel bad.”

##### The Threatening and Exploitative Nature of Cyberbullying

The threatening and exploitative context are other crucial traits that can be acquired by accessing private information, threatening to share it with the public, deleting things that are not theirs, and passing judgment without evidence. For instance, a participant said, “Exploitation using photos or videos or voice messages to request huge amounts of money to remove them is the worst thing.” Another participant said, “Exploitation in public and threatening to reach the relative was so bad.” In contrast, a third one recalled, “Using the private context to request immoral or intolerant things or to threaten the victim was challenging.” Such actions of unauthorized distribution of personal material exacerbated emotional distress and fostered a continual feeling of susceptibility. “They said they would share my pictures if I did not do what they wanted. I felt blackmailed and exploited,” one participant explained.

##### The Public Exposure and Wider Harm of Cyberbullying

Public and remaining possible nature of cyberbullying victimization emerged as a critical challenge to overcome such experiences, which appeared in 3 features, including quick spread, presence of many audiences, and direct psychosocial impacts. “The worst thing about it was happening in the public context and making others laugh at me...” is an example of the many-audience feature. Another participant reported that once harmful content was shared, it spread rapidly across online platforms, inhibiting the ability to move on and recover. “The spread of behavior in social media,” “Even if I delete or deactivate my account, people still remember what was posted,” some participants expressed.

Regarding the direct psychosocial impact of such public experience, when cyberbullying is directed explicitly to the psychosocial effects impacting an individual’s emotional status, social connections, and self-esteem, leading to feelings of inferiority and isolation, because a person tries to avoid others to prevent re-experiencing cyberbullying again. In this regard, participants expressed:

Cyberbullying impacted my self-esteem and caused fear of dealing with others to avoid re-experiencing it again, which impacts all aspects of life, like getting a job.

#### Elements Influencing the Severity of Cyberbullying Experiences

##### Overview

When the participants were asked what they thought were the factors determining the severity of the cyberbullying effects, they mentioned several personal, social, and digital aspects that influence the intensity and duration of cyberbullying impacts.

##### Individual Vulnerability and Coping Capacity

This subtheme includes several individual characteristics, such as age, identity, self-confidence, psychological resilience, prior psychological problems, prior experience of cyber victimization, and lack of awareness of online security and regulations. Participants expressed that younger individuals or those with lower self-esteem are more pointedly affected by cybervictimization. While those with strong emotional regulation were more likely to cope, a participant recalled that “Older people have experience and know how to deal with cyberbullying; younger children are at higher risk.” Further examples that reflect the rest of the characteristics are: “Lack of confidence and weak personality intensify impact,” “being a victim of previous bullying experience.”

The awareness about the response to cyberbullying victimization, like informing about electronic crimes, blocking the bullies, and keeping accounts secure and not giving passwords to anyone, has a great role in strengthening victim response.

##### Role of Others in Shaping the Cyberbullying Experience

This subtheme includes 4 aspects: social agreement, bystander and close people support, unhealthy social relationships and family conflicts, and the relationship with the perpetrator.

Regarding social agreement, participants discuss how cyberbullying begins with one individual and escalates as others join in, forming a larger group engaged in the behavior when the social context does not reject such behavior, leading the perpetrator to repeat the same behaviors due to the nature of social agreement. This quote is an example of “the social agreement, starting with one person, then others participate in the bullying, and it becomes a big group cyberbullying.” Social support arises as a protective factor against the impacts of cyberbullying. A participant said:

Lack of bystander support makes bullies think that they are correct... presence of support, especially emotional support from trustworthy people like family and friends, can minimize cyberbullying impacts.

Those responses indicate that adolescents with supportive families, educators, or peers experienced less anxiety and faster healing.

Unhealthy social relationships and family conflicts worsen cyberbullying impact by adding extra emotional distress. For instance, a participant expressed that “What aggravates the bullying effects is the emotional pressure and the family conflict or problems.” The relationship with the perpetrator was mentioned as an essential factor that interferes with the bullying experience effect. Being close or related vs a stranger can elevate the adverse effects of cyberbullying: “Knowing the perpetrator... the relationship with him, if he is a close friend, relative, or a stranger, differs in the effects.” Additionally, societal expectations regarding shame and reputation frequently prevented teenagers, especially girls, from revealing incidents. “I did not inform my parents since I feared they would confiscate my phone or hold me responsible,” one participant stated.

##### Digital Environment as an Enabler of Cyberbullying

Many participants discussed other digital features that directly determine the severity of cyberbullying’s impacts, including the context of cyberbullying behavior, the tactics used, the purpose of cyberbullying, the number of bullies (one vs group), and the lack of regulations and penalties. Regarding the context of cyberbullying, the type of posted information can aggravate adverse effects because posting personal information to exploit others, sharing sensitive family secrets, or private photos or videos has more emotional impacts than general nonfamily-related information. For instance, a participant stated that “the type of shared information, private versus usual... posting the personal issues online so hurtful.” When it comes to tactics and targets of cyberbullying, the ways used to bully a victim (photos, videos, posts, or comments), the words used, the purpose of bullying (color, body image, or religion), the number of bullies (one vs group), and continuing out of the online context to in-person bullying are essential to determine adverse effects. A participant recalled:

...the ways used to bully victims influence severity of experience, it differs if bullies use images, videos, posts, or comments, and what kind of words are used.

Another participant expressed that:

Targeting the body image or one of the innate characteristics that are difficult to change using hurtful wards... the loneliness effect and realizing that these things that I was bullied about are things that exist in me, and I cannot change them increase the severity of adverse effects on me.

The situation was so terrible because not one but group of my classmates had bullied on me through hurtful wards in the class WhatsApp group, then they started to do the same in the school in front of many.

Some participants mentioned the lack of educational sessions on how to deal with cyberbullying, the lack of protective regulations and rules (eg, no regulation of posts and their spread), and the level of expertise of the perpetrator, all of which are factors that affect how bullying impacts the victim. These quotes illustrate this: “...how expert the perpetrator is influences the impact because of his ability to use different techniques.”

Lack of punishments for bullies, lack of knowledge how to respond, and lack of support for victims... There are no educational sessions on how to deal with cyberbullying.

In conclusion, the study revealed the multifaceted nature of adolescents’ experiences with cyberbullying. The findings highlight that beyond the digital realm, cyberbullying effects extend to affect psychological status and social functioning, including emotional distress, anxiety, cognitive disturbances, social withdrawal, and damaged trust. Key challenges in coping with cyberbullying were linked to the anonymity of perpetrators, the exploitative use of private information, and the public nature of online attacks, all of which intensified feelings of helplessness and humiliation. The presence of social support is a protective factor that facilitates recovery from cyberbullying victimization. The severity of these effects was influenced by personal vulnerability, social agreement and support, the nature of the relationship with the perpetrator, and digital factors such as the tactics used, the number of bullies involved, and the absence of regulations or accountability mechanisms. Together, these findings highlight the urgent need for targeted prevention, context-sensitive interventions, and educational initiatives to promote emotional resilience and online safety among Jordanian youth.

## Discussion

### Overview

This study added evidence on the effects of cyber victimization and elaborated further on the challenges and determinants of this experience. The results can be interpreted via the Stress–Diathesis Model, which suggests that psychological outcomes result from the interplay between environmental stressors and personal vulnerabilities [21,22]. Cyberbullying served as a major stressor in this study due to its ongoing presence, anonymity, public visibility, and exploitative nature. Participants’ experiences vary based on factors such as individual coping abilities, resilience, and prior emotional vulnerabilities. Participants with limited coping resources seemed more vulnerable to anxiety, emotional suffering, and social isolation. Concurrently, social support from family, peers, and teachers served as a protective factor, mitigating the adverse psychological effects. These results indicate that the effect of cyberbullying is influenced not only by exposure but also by the complex interplay of digital stressors and personal vulnerabilities.

The findings provided an answer to the first research question about the effect of cyberbullying experience. According to the participants, cyber victimization mainly affects psychological health and social relationships, which are sometimes overlapping. This is in line with Sung [41], who states that cyberbullying impacts psychosocial adjustment, including mental health, self-esteem, and social relationships. The negative emotions that cyberbullying elicited ranged widely from wanting to avenge and retaliate to feeling sad, hurt, embarrassed, angry, humiliated, isolated, marginalized, and powerless. The findings are also consistent with a study that used data from a qualitative thematic analysis of several data sources considering the effects of cyberbullying; numerous people mentioned crying for extended periods and feeling upset, emotional, choked, crushed, hurt, and generally miserable [42].

Cyberbullying victimization as a stressful situation may lead to changes in the brain’s internal structures, as shown by the discovery that cyberbullying victims have more severely disrupted cortisol reactivity [20] and altered emotional brain function [43]. Social isolation is one of the most severe effects of cyberbullying. It differs significantly from traditional bullying in that there are no limitations on when or where technology can be used for communication. Social isolation, increased suspicion, and signs of posttraumatic stress disorder may happen when cyberbullying tactics expose the victim’s humiliation to an extensive audience viewing [44]. The lack of acceptance among their peers causes victims to feel lonely and socially isolated, have low self-esteem, and be depressed [45,46]. It can also cause stress-related disorders, problems with concentration and learning in school, emotional disorders, and even suicidal thoughts [46]. Thus, developing effective prevention and intervention strategies to support those affected is crucial.

The analysis supported the second question that participants’ narratives revealed persistent barriers to overcoming cyberbullying victimization. Participants shared a wide range of thoughts on the most challenging part of their cyberbullying experience. They described those difficulties in a manner that reflects the unique cyberbullying attributes of anonymity, publicity, and threatening context. Cyberbullying possesses distinctive traits due to its online nature and lack of face-to-face contact, such as its persistent nature, broad audience reach, and anonymity, distinguishing it from traditional bullying [47]. Anonymity, particularly in public online spaces, may hinder prevention efforts as perpetrators can remain unidentified. Anonymity exacerbates feelings of helplessness and perceived power imbalances in cyberbullying situations, pushing victims to keep their experiences silent without reporting. Victims may feel powerless and vulnerable when perpetrators possess embarrassing or harmful material that cannot be controlled and published online at any time in front of many people [48]. However, earlier studies, predating 2010, did not prioritize these characteristics, as evidenced by the systematic review by Berne et al [49].

Participants stated that the threatening and exploitative context of cyberbullying is one of the critical reasons why they consider this experience challenging, primarily when the bully uses private information to request immoral or intolerable things or to threaten the victims in public and threaten to reach their relatives. Also, accusing victims and passing judgment on them for things that are not true without evidence leads to feelings of inadequacy and altered psychosocial functioning, including suicidal thoughts and avoidance of social interaction to avert re-experiencing bullying. Thus, it is necessary to develop interventions to overcome such situations. For example, using celebrity endorsements is effective in highlighting the effects of cyberbullying and favoring an inclusive strategy that educates on prevention skills, promotes reporting, allows anonymous online reporting, and revokes internet privileges for perpetrators, where mandatory reporting, suspensions, or police involvement predicted a significant decrease in support from uninvolved students, witnesses, victims, and perpetrators [50].

The study findings illustrated that personal and interpersonal factors significantly shape how severe the impact of cyberbullying is on adolescents, which answers the third question. Adolescents in Jordan stated that the severity determinants of experience impacts are an integration of many personal, social, and digital factors related to cyberbullying behavior. According to participants, with age, people gain more skills and knowledge to deal with bullying; personality and beliefs, psychological strength, awareness of online security, and previous bullying experience are the most personal determinants of cyberbullying effects. Subsequently, younger children need more interventions that focus on enhancing their awareness of and skills to deal with cyberbullying and online websites, specifically school-based intervention programs [51] that target emotional intelligence and strengthen psychological well-being.

The participants stated that some social factors determine the severity of cyberbullying effects as well, such as social acceptance, lack of support, family conflicts, and relationship with the bully. Given the reality that adolescents experience marked patterns of cyberbullying victimization and perpetration based on individual and social support factors [52]. Social support is essential for victims of cyberbullying because it can protect them from the damaging effects of bullying. Individuals are more able to handle the strain and emotional anguish brought on by cyberbullying when they have supportive relationships with friends, family, or peers [53]. In addition to offering practical help, emotional validation, and a sense of community, social support can make victims feel less alone and more resilient in the face of bullying [54]. Having supportive relationships can also motivate victims to report incidents, seek assistance, and take precautions to stay safe online when they believe they have friends who support them in solving problems, regaining their confidence, and resuming social interactions in their surroundings [55]. In general, social support is essential for reducing the negative impacts of cyberbullying and enhancing the well-being of its victims. However, there is low perceived family and peer social support among youth [56], making this an essential area for intervention. As youth spend a long time online, initiatives are needed to promote online help-seeking and empower parents and peers to support their well-being [57].

Lastly, participants talked about some digital factors that are directly related to the nature of cyberbullying behavior, which were essential in determining the severity of victimization. The type of posted information can aggravate adverse effects because posting personal information to exploit others, sharing sensitive family secrets, or sharing private photos or videos has a greater emotional impact than general, nonfamily-related information. On the other hand, the ways used to bully victims (photos, videos, posts, or comments), the words used, the target of bullying (color, body image, or religion), the number of bullies (one vs group), and continuing out of online context to in-person bullying are essential determinants of adverse effects.

### Implications

The findings of this study underscore the urgent need for culturally responsive victim-centered clinical and technology supported interventions, policy reforms, and accessible mental health services to mitigate the psychological and social harms of cyberbullying on adolescents. Raising awareness of harmful content and the dangers of sharing personal information online should be prioritized through school-based education and parental guidance. Schools need targeted interventions, such as implementing digital safety and mental health programs that include emotional regulation training and peer-support methods, in light of the identified effects of distress, isolation, and relationship difficulties, taking into consideration the sociocultural context of adolescents and parents, such as communication norms and culturally specific views on online conduct. Family-based interventions are essential, as parents play important protective roles. These interventions should focus on open communication, digital literacy, and supportive oversight methods that can enhance teenagers’ ability to cope and their resilience when dealing with cyberbullying. Mobile apps or online platforms offering psychoeducational resources could enhance parental understanding and facilitate early recognition of distress related to cyberbullying, effectively.

Health care providers, especially nurses and public health professionals, have a critical clinical role in routine screening for bullying during medical visits, such as including questions on intake forms [51], which can facilitate disclosure and early intervention. Following the COVID-19 pandemic, increases in online activity have altered the landscape of cyberbullying, highlighting the need for updated, trauma-informed care policies [58]. Moreover, integrating innovative tools, such as narrative-based audio-visual interventions, into prevention programs may enhance adolescents’ engagement and emotional processing. For instance, initiatives like programs that have shown that multimedia storytelling can foster empathy and resilience [59,60]. In line with scalable digital mental health strategies [61], interventions could benefit from using mobile health technologies, digital counseling services delivered through schools, and chatbot-assisted psychoeducation to deliver prompt, accessible assistance. Future studies should be built on longitudinal, intervention-based designs to explore the efficacy and cultural relevance of these technology-driven approaches across various social and cultural contexts.

### Limitations

Although this study provides valuable insights into the contexts and perspectives surrounding cyberbullying victimization among adolescents in Jordan, several limitations should be acknowledged. First, data collection relied solely on 3 open-ended narrative questions within a survey, which may have limited the depth and nuance of participants’ responses in reflecting subtle coping strategies, long-term effects, or intricate family and school interactions. Future research could benefit from using in-depth interviews or focus groups to allow adolescents’ experiences to be expressed more richly and in greater detail. Second, the sample was drawn exclusively from central and northern Jordan, limiting the generalizability of the findings to adolescents in other regions. Environmental, cultural, and technological factors in southern or rural areas may shape cyberbullying experiences differently.

Additionally, the use of self-report methods may introduce bias due to participants’ desire to present themselves in a favorable light or their reluctance to disclose sensitive information. The timing of data collection during the school year may also have influenced responses, as experiences of cyberbullying can fluctuate depending on school-related stress and peer interactions. To strengthen generalizability and validity in future research, studies should aim to include more diverse regional samples, consider longitudinal designs to capture temporal patterns, and incorporate mixed methods approaches that combine narrative inquiry with quantitative measures. Future studies should also explore how gender may shape experiences, interpretations, and coping strategies related to cyberbullying.

### Conclusion

This study provides a novel and context-specific contribution to the understanding of cyberbullying’s psychological and social impact on adolescents in Jordan, a region where such research remains limited. Our findings illuminate how the anonymity of perpetrators, the exploitative nature of online threats, and the public exposure of cyberbullying significantly amplify adolescents’ emotional distress, social withdrawal, and behavioral disruption. Notably, the results also highlight how the absence of moral courage and bystander intervention, shaped by social dynamics, personal vulnerabilities, and unhealthy relationships, intensifies victims’ feelings of isolation and helplessness. This underscores the need to incorporate frameworks that foster moral courage and active defense behaviors into intervention strategies, which are currently underexplored. By addressing these gaps, this study contributes to a victim-centered understanding of cyberbullying and offers direction for targeted, culturally relevant prevention efforts. Future research should build on these insights by using in-depth interviews or longitudinal designs to explore long-term effects and associated outcomes such as coping mechanisms, academic disruption, self-harm, and other high-risk behaviors. These extensions would deepen our understanding of how early experiences with cyberbullying shape adolescent development and inform more holistic and trauma-informed support systems.

## Abbreviations

RCBI-II: Revised Cyber Bullying Inventory–II
